# Supplementary material for: Glycosylation Analysis of Urinary Peptidome Highlights IGF2 Glycopeptides in Association with CKD
Source: Int J Mol Sci. 2023 Mar 11;24(6):5402. doi: 10.3390/ijms24065402 (PMC10048973; doi:10.3390/ijms24065402)
Supplement: Supplementary file 1 [file ijms-24-05402-s001.zip › Supplementary Information.pdf]

Supplementary Information Index

Table S1 – Proteome Discoverer 1.4 output of protein identifications for all 10 CE-MS/MS raw files

Table S2 – Proteome Discoverer 1.4 output of peptide identifications for all 10 CE-MS/MS raw files

Table S3 - Urinary nine IGF2 peptides

Table S4 - Results of correlation analyses

Table S5 – Results of ELISA assay

Table S6 – Outputs from "prediction tool" Proteasix
